# Supplementary material for: “If it’s necessary, it has to be done. And that’s for the physician to decide, not me.” Imaging techniques in monitoring routines in coronary heart disease and post-stroke patients: A qualitative interview study from the patients’ perspective
Source: PLoS One. 2025 Dec 9;20(12):e0338431. doi: 10.1371/journal.pone.0338431 (PMC12688143; doi:10.1371/journal.pone.0338431)
Supplement: S2 Text — (DOCX) [file pone.0338431.s002.docx]

**Interview Guide – Patients with Coronary Heart Disease (CHD)**

**Project Aim:**
To explore how patients with stroke or coronary heart disease experience and evaluate monitoring routines such as echocardiography and carotid duplex sonography.

**Introduktion**

Thank you very much for taking the time to participate in this interview today.

We would like to speak with you about your experience with coronary heart disease, and are particularly interested in how you perceive the follow-up examinations and monitoring practices conducted by your physicians.

I am […] and work as […]. Please note that I am not a physician.

I will guide you through a set of open-ended questions. The conversation will last approximately 30 to 45 minutes. Please feel free to take your time, request a break, or end the interview at any time if you wish.

**Warm-up**

Did you get here comfortably? / Did the technical setup work for you?

What are your expectations for this interview?

**Medical History**

Let’s begin with your personal story.

Could you please tell us, in your own words, how your first symptoms started and when the diagnosis was made?

Please speak freely – I may take some notes and ask follow-up questions afterwards.

**1. Regular monitoring routines after diagnosis of CHD**

- Have you received regular follow-up examinations since your heart attack or diagnosis of CHD?
- Which physicians are involved in your follow-up care?

**1.1 GPs**

- What types of examinations are performed during your GP visits?
- How frequently are these examinations conducted?
- Could you describe how a typical consultation takes place?
- Do you recall what questions your GP typically asks?
- Are physical examinations performed? If so, what do they involve?
- Are any of these examinations self-paid? How do you feel about that?

***If echocardiography is performed in general practice:***

- Do you usually request the echocardiography yourself, or is it initiated by the physician?
- How are appointments scheduled?
- At what intervals is the echocardiography performed?
- Who determines the frequency?
- How do you feel about the interval between appointments?
- Does undergoing the duplex sonography trigger any emotional reactions for you?
- Are you shown and explained what is seen during the imaging?
- How would you feel if these examinations were no longer conducted?
- And what if other procedures, such as blood tests, were discontinued?
- What benefits do you associate with these examinations?
- Do you have any suggestions for improving the process?
- Do these check-ups sometimes lead to referrals to specialists or hospitals?
  - What is your opinion on such referrals?

**1.2 Specialist physicians – e.g. cardiologists**

- What types of examinations are performed during your specialist visits?
- How frequently are these performed?
- How does a typical consultation proceed?
- What kind of questions do they ask?
- Are you physically examined? If so, how and how often?
- Do you have to pay for any of these tests yourself? How do you feel about that?

***If duplex sonography is performed by a specialist physician:***
(Same set of questions as above under the GP section)

**1.3 Additional specialist physicians**
(Repeat the above structure for any other specialist involved in care.)

**Visual Elicitation (Photo Cards)**

When you think about your heart attack or CHD, which image best represents your emotional experience?

- Why?

And when you think about the regular monitoring examinations, which image represents your feelings best?

- Why?

(If not mentioned before): Does this image also reflect how you feel during the echocardiography?

**Mental Health & Emotional Impact**

- Have you ever been, or are you currently, receiving psychological support due to your chronic condition?
- Did you seek psychological support on your own initiative or was it recommended by a physician?

**Perception of monitoring**

- Do you feel well cared for through your regular follow-up examinations?
- Do they give you a sense of safety or help to reduce the fear of having another cardiac event? Why or why not?

Imaging techniques such as echocardiography allow us to see inside the body.

- - Is this visualisation important to you?
  - Does it help you understand your condition better?
  - Or do you think it gives your physician a clearer picture?

**Closing**

Is there anything else you would like to share that we haven’t addressed yet?

Thank you very much for your time and for sharing your insights. If you wish, we would be happy to inform you about the results of the study.

**Interview Guide – Patients Post-Stroke**

**Project Aim:**
To explore how patients with stroke or coronary heart disease experience and evaluate monitoring routines such as carotid duplex sonography and echocardiography.

**Introduktion**

Thank you very much for taking the time to participate in this interview today.

We would like to speak with you about your post-stroke experience and are particularly interested in how you perceive the follow-up examinations/monitoring routines conducted by your physicians.

I am […] and work as […]. Please note that I am not a physician.

I will guide you through a set of open-ended questions. The conversation will last approximately 30 to 45 minutes. Please feel free to take your time, request a break, or end the interview at any time if you wish.

**Warm-up**

Did you get here comfortably? / Did the technical setup work for you?

What are your expectations for this interview?

**Medical History**

Let’s begin with your personal story.

Could you please tell us, in your own words, how your first symptoms started and when the diagnosis was made?

Please speak freely – I may take some notes and ask follow-up questions afterwards.

**1. Regular monitoring routines after stroke**

- Have you received regular monitoring since your stroke?
- Which physicians are currently involved in your follow-up care?

**1.1 GPs**

- What types of examinations are performed during your GP visits?
- How frequently are these examinations conducted?
- Could you describe how a typical consultation takes place?
- Do you recall what questions your GP typically asks?
- Are physical examinations performed? If so, what do they involve?
- Are any of these examinations self-paid? How do you feel about that?

***If duplex sonography is performed in general practice:***

- Do you usually request the duplex sonography yourself, or is it initiated by the physician?
- How are appointments scheduled?
- At what intervals is the duplex sonography performed?
- Who determines the frequency?
- How do you feel about the interval between appointments?
- Does undergoing the duplex sonography trigger any emotional reactions for you?
- Are you shown and explained what is seen during the imaging?
- How would you feel if these examinations were no longer conducted?
- And what if other procedures, such as blood tests, were discontinued?
- What benefits do you associate with these examinations?
- Do you have any suggestions for improving the process?
- Do these check-ups sometimes lead to referrals to specialists or hospitals?
  - What is your opinion on such referrals?

**1.2 Specialist physicians – e.g. neurologist**

- What types of examinations are performed during your specialist visits?
- How frequently are these performed?
- How does a typical consultation proceed?
- What kind of questions do they ask?
- Are you physically examined? If so, how and how often?
- Do you have to pay for any of these tests yourself? How do you feel about that?

***If duplex sonography is performed by a specialist physician:***
(Same set of questions as above under the GP section)

**1.3 Additional specialist physicians**
(Repeat the above structure for any other specialist involved in care.)

**Visual Elicitation (Photo Cards)**

When you think about your stroke or TIA, which image best represents your emotional experience?

- Why?

And when you think about the regular monitoring examinations, which image represents your feelings best?

- Why?

(If not mentioned before): Does this image also reflect how you feel during the duplex sonography?

**Mental Health & Emotional Impact**

- Have you ever been, or are you currently, receiving psychological support due to your chronic condition?
- Did you seek psychological support on your own initiative or was it recommended by a physician?

**Perception of monitoring**

- Do you feel well cared for through your regular follow-up examinations?
- Do these provide a sense of security or help reduce your fear of having another stroke? Why or why not?

Imaging techniques such as duplex sonography allow us to see inside the body.

- - Is this visualisation important to you?
  - Does it help you understand your condition better?
  - Or do you think it gives your physician a clearer picture?

**Closing**

Is there anything else you would like to share that we haven’t addressed yet?

Thank you very much for your time and for sharing your insights. If you wish, we would be happy to inform you about the results of the study.
