# Supplementary material for: “If it’s necessary, it has to be done. And that’s for the physician to decide, not me.” Imaging techniques in monitoring routines in coronary heart disease and post-stroke patients: A qualitative interview study from the patients’ perspective
Source: PLoS One. 2025 Dec 9;20(12):e0338431. doi: 10.1371/journal.pone.0338431 (PMC12688143; doi:10.1371/journal.pone.0338431)
Supplement: S3 Table — (DOCX) [file pone.0338431.s003.docx]

| **List of Codes** | | |  |  |
| --- | --- | --- | --- | --- |
|  | **Code** | **Subcode** | **Definition** | **Anchor Quotes** |
|  | **Omission of ultrasound or reduction of ultrasound frequency** | | | |
|  |  | Ultrasound is somewhat important | Feeling of importance, uneasiness, and disappointment about the lack of ultrasound | *I might not say anything at first. But then I would talk to my family doctor and say that I have some uneasiness there. (P-13) So first of all, for the ultrasound examination, I would say that if the patient was given the option of saying with or without Doppler for an extra charge or whatever, then I would opt for it, so fine. (P-03)* |
|  |  | Ultrasound is unimportant | Lack of interest in the examination | *So, if he thinks it’s necessary, then he has to do it. I have no interest in the examination. (P-02)* |
|  |  | Loss of control | Feeling of loss due to lack of ultrasound | *I would think / yes, of course, you lose a bit of control. You don’t know what’s going on. (P-01)* |
|  | **Experience of the ultrasound itself** | | | |
|  |  | Not important | No special emotion or significance of the ultrasound | *So, if he thinks it’s necessary, then he has to do it. I have no interest in the examination. (P-02)* |
|  |  | Protection | Protection from recurrence | *I don’t know about protection. No, not really. But safety in the sense that nothing is happening at the moment. Protection is nonsense, because it could be different tomorrow, right? (P-14)* |
|  |  | Safety | Feeling of safety through ultrasound examinations | *Well, but as I said, you just have more safety, right? You feel safer, I think, actually, yes. (P-01)* |
|  |  | Precaution | Feeling of prevention through ultrasound | *Also, ahead of new events. Because I think that helps me, and that things happen less often, and so on. And I think that’s a positive thing. (P-02)* |
|  | **Experience of**  **monitoring in the setting** | | Description of the entire monitoring process as being in good hands, satisfied, reassuring, and informative, but also accusatory/investigative | *And yes, so he doesn’t just do his usual routine and says: "Bye, take care, see you next time", he also says: "We’ll take a closer look", and/or that, so I have the impression that they care and things are going as they should. (P-05)* |
|  | **Perception of the specialist from the patient’s perspective** | | | |
|  |  | ... as a distant practitioner | Feeling of not being taken seriously, the patient is not seen as an individual | *He isn’t very empathetic, is he? He reels it off, and yes, and now you do this, now you do that and this, and finally he says: “Yes, it works.” (P-13)* |
|  |  | ...as first line of treatment | Specialist is the first point of contact | *I usually bring it up [with the GP], at least if it’s close in time, that I’ve just been to the check-up beforehand, that I was there. And the specialist or cardiologist was satisfied, and so was I. Then he says: “Yes, we’ve received the report.” Then that’s usually enough. (P-03)* |
|  |  | ...as a friend | Physician is privately known or known for a long time, amicable relationship | *It’s such a large internal medicine practice, relatively mass-produced. But the advantage is that the nephrologist is from [big city, 448 km away] (laughs). I grew up in [big city, 448 km away]. And the funny thing is, I had a completely different connection with him, right? We talked about [soccer club]. (P-13)* |
|  |  | ...as a guardian | Physicians look after their patients | *Well, I feel that someone cares about how I’m doing. And that’s why I think it’s important, and I think it helps me. (P-02)* |
|  |  | ...as an expert | Physician is characterised by special expertise | *That’s why I think that a GP has to go in that direction, in management and the rough stuff. As soon as it gets more delicate, you have to send it to a specialist. (P-10)* |
|  | **Perception of the GP from the patient's perspective** | | | |
|  |  | ... as a distant practitioner | Feeling of not being taken seriously, GP shows a lack of interest in visits to specialists | *...from next year, I will be quite elderly. Sometimes you get the feeling that some people don’t take it seriously any more. The way doctors say: “What do you actually want? What do you actually expect?” (P-13) I always give him my reports. He says: “I don’t look at them. I trust the specialists.” (P-10)* |
|  |  | ...as first line of treatment | GP as the first point of contact | *And if there were any issues, I could always go there without any problems. In that sense, it was quite good as a first point of contact. (P-06)* |
|  |  | ...as a service provider | Get referrals or prescriptions, and have blood tests done for the specialists | *In other words, he / I / when I had the appointment with the cardiologist in front of me, I went to the family doctor about / the week before and had this blood test with the breakdown into the different blood lipid variations done so that he could take these values into account. (P-04)* |
|  |  | ...as a friend | Physician is privately known or known for a long time, amicable relationship, mutual trust | *But as I said, Mrs. [family doctor] relies on what I say, so to speak. And that works. (P-14)* |
|  |  | ...as a guardian | GP takes care for all concerns, takes an interest; patient feels well looked after | *Because I think I’m in good hands with my GP. He cares a lot about what we do. He examines me and also gives me advice. He also has a say in my medication. And I think I’m well managed. (P-02)* |
|  |  | ...as coordinator | GP as manager, has an overview; good coordination between GP and specialist, GP discusses specialist reports | *As such, the GP has everything under control. (P-02)* |
|  |  | ...as an expert | GP has special knowledge | *He has an ECG machine. I also had a long-term ECG done by them, and so on. That’s also good if he knows his stuff. Yes, it just depends on where it is then / but he has to recognise when it becomes more critical. Well, I trusted my family doctor. (P-10)* |
|  | **Motives for monitoring** | | Motives for examinations from the patient’s perspective | *I feel that I am in good hands. And I understand that they always have to check this. I think that’s fine. (P-07)* |
|  | **Education** | | How patients are informed about monitoring | *However, I also had the other experience that I didn’t actually have to ask because the doctor really explained everything to my satisfaction. At the latest, then in the follow-up consultation. (P-03)* |
|  | **Obtaining information**  **through ultrasound**  **(imaging)** | | What patients learn through imaging | *Yes, he just says, okay, pretty well healed. [...] You can’t see or notice anything anymore. (P-01)* |
|  |  | Visualisation for the physician | The importance of visualisation for the doctor from the patient’s perspective | *I think so, definitely. I mean, physical examination, yes. But this imaging, yes, it’s more accurate, isn’t it? (P-01)* |
|  | **Consequences of**  **monitoring** | |  |  |
|  |  | Frequency adjustment | The frequency is adjusted in the course of monitoring | *And I have also had this done by this internist here about every three years. He then reassured me and said that the condition is roughly the same. Every five years is enough, but that’s what we did. (P-03)* |
|  |  | Referral | Referrals for cardiac catherization, to the hospital for further examinations, for surgery | *He said that they would do a catheter, but if they don’t find anything, it could still happen in the following three weeks. Then I said: “Should we just leave it?” “No, that’s important.” (P-13)* |
|  |  | Medication adjustment | Changes in medication as a consequence of monitoring | *Nothing. He just keeps tinkering with the tablets, sometimes a little more, then sometimes he takes some away again. (P-01)* |
|  | **Wishes for the (own)**  **care structure** | | What patients want from their entire medical care system | *And when you get older, it would perhaps make sense if you could go somewhere once every three months and then you’d just get through in three or four hours and be done, right? (P-01)* |
|  | **Perception of (own)**  **care structure** | | Patients feel as being in good hands despite long waiting times for specialist appointments | *So the time it takes to get an appointment, but otherwise I feel pretty well looked after medically. (P-09)* |
|  |  | Perception of undersupply | Perception of underuse due to structural problems | *And what I find bad is that in most cases / with some physicians, you can’t get an appointment. Sometimes you have to wait half a year before you can even get in. (P-02)* |
|  |  | Oversupply | Perception of excessive care due to too many unnecessary examinations | *And everything is done twice, yes? Many examinations are done both here and there. Why is that? Yes? They are / Well, I don't understand that. And you could save a lot of money without compromising quality. (P-06)* |
|  |  | Time | Time as a resource that is needed to be able to attend appointments related to the illness. | *And I now have, if I show you the calendar, every day I virtually drive him to the eye doctor, ENT doctor, neurologist, physiotherapist, and speech therapist. (P-02)* |
|  | **Responsibility** | |  |  |
|  |  | Physician’s responsibility | Responsibility for health is seen as lying with the physician | *Well, the way I see it, if the doctor does it (...) then that / if he thinks it’s necessary, then I think I have to go there. (...) Because he does it indirectly for me or directly for me. (...) Then I go there. (P-02)* |
|  |  | Empowerment | Self-determination; explanations or examinations are requested or rejected; self-management | *And then I have to say to the doctor: “Have you checked the LDL value and sugar?” (P-13)* |
|  | **Dealing with the condition today** | |  |  |
|  |  | Discomfort | The condition still causes fear or discomfort today. | *Discomfort. So fear or worry about what will happen. (P-13)* |
|  |  | Carefree | The event is in the past and has less influence today. | *No, I actually feel quite well. (P-09)* |
|  | **Experiences directly**  **after diagnosis/event** | | Expectations of oneself, the medical and social environment, as well as the disease and its course in the future. | *I thought it would knock you out now. But I have to be honest, that was one or two weeks, and then I was relatively well again. (P-01)* |
|  | **Importance of**  **comorbidities** | | Importance and influence of other existing illnesses. | *I have another problem [than the stroke]. When I make an effort to think now, I have this pain. I try ... and when I concentrate, I get exactly the same pain. Always in the same region. (P-10)* |
|  |  | Burden of examinations | Frequency of examinations for comorbidities and multimorbidity. | *But because he has a neurologist in [city, 11 km away], a diabetologist in [large city, 12 km away], the other one is here in [place of residence], the third one is in [large city, 18 km away], the urologist, I don’t get anything. (P-02)* |
|  | **Focus on diagnosis** | | Importance and relevance for the interviewees of receiving a diagnosis in the course of their medical history. | *[What bothers him is] that no cause in the sense of the blocked artery was actually found, yes. (P-12)* |
|  | **Treatment frequency** | | At what intervals do treatments take place that are related | *Yes, I see the doctor [cardiologist] every six months. (P-01)* |
|  |  | Monitoring frequency | At what intervals monitoring examinations take  place (duplex ultrasound, cardiac ultrasound) | *Once a year, there was only an examination with ultrasound and ECG. In addition, the second examination per year included an exercise ECG and scintigram. (P-04)* |
|  |  | Perception of frequency | Personal perception of whether the frequency of treatment and monitoring is too high, too low or appropriate. | *Well, at the moment I’m satisfied with the annual cycle. (P-03)* |
|  | **Influence of relatives** | | Emotions or actions of relatives that influence the interviewee’s perception or behaviour in relation to the disease and perception of appointments. | *Then my son said, “Well, mom, I mean, you shouldn’t do it [take the novel drug].” (P-07)* |
