# Supplementary material for: “If it’s necessary, it has to be done. And that’s for the physician to decide, not me.” Imaging techniques in monitoring routines in coronary heart disease and post-stroke patients: A qualitative interview study from the patients’ perspective
Source: PLoS One. 2025 Dec 9;20(12):e0338431. doi: 10.1371/journal.pone.0338431 (PMC12688143; doi:10.1371/journal.pone.0338431)
Supplement: S4 Table — (DOCX) [file pone.0338431.s004.docx]

| **Theme** | | | **Subtheme** | | |
| --- | --- | --- | --- | --- | --- |
| **Title** | **Definition** | **Anchor Quote(s)** | **Title** | **Definition** | **Anchor Quote(s)** |
| Perception of a high-quality of care, which is organisationally challenging | The healthcare system is perceived with mixed feelings, though patients feel well cared for despite long waiting times or assumed medical over- or underuse. | “[...] it’s always a bit problematic in the healthcare system to get an appointment with some specialists [...] Well, I know. I just have to give six months’; notice, that’s okay.” (P-11) | GPs as guides in patient care | GPs are seen by patients as the central point of coordination in their care. | “[...] I am in the opinion that a GP has to go in that direction, in management and the rough stuff.” (P-10) |
|  |  |  | Cardiologists and neurologists as specialised experts in patient care | Neurologists and cardiologists are seen by patients as specialists who are consulted with complex problems. | “Just like everywhere else in the profession, you need specialists who can do this. Then it’s quicker and more accurate.” (P-10) |
| Monitoring takes place, but patient engagement varies | Monitoring takes place regardless of the extent to which patients deal with their illness and question their medical care. | “And if necessary, I also asked about it, so I already knew what it was about. And I also asked what the measurement was, what the value was and what range it was in, depending on the situation.” (P-03) “Yes, just as I said, if it’s necessary, it has to be done. And that’s for the physician to decide, not me.” (P-02) |  | | |
| Positive appraisal of the monitoring frequency | Patients feel that the frequency of the monitoring routines is appropriate and tailored to their individual disease situation. | “No, it’s enough for me. And if I had the impression that I needed more, then that would certainly not be a problem.” (P-14) |  | | |
| The perceived benefits of monitoring influence the experience and perception of these examinations | Patients with regular monitoring routines attribute a positive benefit to monitoring and experience imaging procedures such as duplex sonography or echocardiography as a pleasant examination method. | “Well, there is something reassuring when you hear that the plaque formation has either not progressed any further [...] or if it is found that there is perhaps only 20 percent flow and a stent would be necessary.” (P-03) | Monitoring provides safety, while hypothetical discontinuation of monitoring faces concerns | Many patients who receive regular check-ups would perceive a discontinuation as a significant cut. Elimination is associated with rationing and often leads to uncertainty about the remaining quality of their own care. | “[Elimination of imaging check-ups] No, that wouldn’t actually be okay with me. I think it’s important to have this additional certainty that nothing significant has deteriorated.” (P-12) |
| Monitoring examinations may have therapeutic consequences | Therapeutic consequences of medical consultations can be, for example, the adjustment of medication or the issuing of referrals. Whether these consequences occur directly as a result of ultrasound scans is unclear. | “[...] In this respect, I am reassured that the next step was taken as a consequence of this examination in the practice. So I am also happy that this could be done so quickly.” (P-06) |  | | |
