# Supplementary material for: “If it’s necessary, it has to be done. And that’s for the physician to decide, not me.” Imaging techniques in monitoring routines in coronary heart disease and post-stroke patients: A qualitative interview study from the patients’ perspective
Source: PLoS One. 2025 Dec 9;20(12):e0338431. doi: 10.1371/journal.pone.0338431 (PMC12688143; doi:10.1371/journal.pone.0338431)
Supplement: Attachment — Submitted filename: Response to Reviewers.pdf [file pone.0338431.s006.pdf]

Review Comments to the Author

Reviewer #1:

Introduction - please include a sentence or two about how CHD and stroke patients share characteristics. Please explain that CHD can be a risk factor for stroke, so interviewing both cohorts could help with longitudinal monitoring to predict changes before stroke, in the case of patients with CHD only. Please include information about the differences between stroke and TIA, since TIA patients were included in this analysis. TIA is also a risk factor for stroke, so it should be considered the way CHD is - a potential diagnosis to monitor to prevent a stroke.

Response: Thank you for your thoughts on this. Both conditions have been chosen as examples for monitoring routines. In this context, it was considered whether the conditions are relevant to primary care and whether the monitoring routines can be reflected in billing data for another part of the project. From our perspective, there was no thematic connection between the two conditions. Therefore, although you were absolutely right to describe these connections, we would not include them in the introduction, as they were not part of the study question and methodology.

Did your patients with TIA also participate in regular echocardiography and carotid duplex sonography monitoring? What does "regular monitoring" intervals mean, i.e. how frequent? Were these procedures performed in a clinical setting or at home?

Response: Yes, all patients included in our study had undergone at least three echocardiography or carotid duplex sonography examinations within the last three years. That was one of the recruitment criteria. Monitoring intervals ranged between every three months and every eighteen months (see RESULTS – Theme 3). All examinations were performed in outpatient clinical settings by cardiologists or neurologists.

Changes in manuscript:

- METHODS “Participants included adult patients diagnosed with CHD, stroke or TIA who had undergone echocardiography or carotid duplex sonography monitoring at least three times within the past three years.”
- RESULTS “The participants reported that echocardiography or carotid duplex sonography monitoring is carried out exclusively by cardiologists and neurologists in an outpatient clinical setting, not in GP practices.”

Methods - please provide a paragraph explaining the rationale for why reflexive thematic analysis by Braun and Clarke is best suited for this project. Define this analysis approach for the study.

Response: A paragraph defining and justifying the use of reflexive thematic analysis has been added to the Data Analysis section. This addition clarifies the flexibility and reflexivity of this method and its alignment with our research aims.

Changes in manuscript: “The qualitative data analysis followed the thematic framework by Braun and Clarke (18, 19), as it is a flexible analytic approach that allows researchers to identify, analyse, and interpret patterns of meaning across qualitative data. It emphasises researcher reflexivity and the active role of interpretation. This method was particularly suited to our study because it enabled both inductive and deductive exploration of participants’ experiences and perceptions of monitoring without being bound to a predefined theoretical framework, by following a systematic process of data immersion, coding and theme development.”

Participants and recruitment - how were diagnoses defined? Did you obtain diagnosis from electronic medical records or Patient self-reporting?

Response: Diagnoses were based on patient self-reporting during recruitment. As the study focused on patients' experiences rather than clinical validation, self-reported diagnoses of CHD, stroke, or TIA were considered appropriate.

Changes in manuscript: "Diagnoses of CHD, stroke, or TIA were based on patient self-reporting during recruitment."

Data analysis - Could you start this section with the sentence so readers know which tool was used: "The analysis was conducted using MAXQDA (2020) software."?

Response: We agree and have moved this sentence to the beginning of the data analysis section.

Results - could you create a participant enrollment diagram that shows how many patients you contacted, the 29 who replied (and how they dropped out), the 14 who completed the study, and then 2 separate boxes showing the number of study completion participants by diagnosis (CHD diagnosis vs stroke diagnosis)?

Response: We appreciate this suggestion. However, it was not possible to determine the total number of individuals reached by our recruitment efforts, as participants were recruited a public press calls and several general practices. Therefore, we could not create an enrollment flowchart.

We clarified in the Results section how participants were selected and why some were not included, describing the screening criteria and selection process in more detail.

Changes in manuscript: "Of these, 14 interviews were scheduled and conducted, comprising individuals with diverse backgrounds in terms of age, gender, disease duration and time of response. Individuals who had not yet undergone, or had completed only one monitoring examination, were excluded during screening, as the study focused on patients with regular monitoring".

Could you give the average/mean for this: "lasted between 20 and 51 minutes."?

Response: Thank you for the suggestion. We added the mean interview duration to the Results section.

Changes in manuscript: "The interviews took place between November 2023 and March 2024 and lasted on average 32 minutes (range 20-51 minutes)."

Is there any information about participant education levels? Do you have ethnicity information?

Response: As the study focused on patients' experiences rather than sociodemographic differences, education and ethnicity data were not collected.

Line 222 is missing a leading " for the quote.

Response: Thank you for catching this. The missing quotation mark in line 222 has been added.

Discussion - after line 281, you mention "The results of our study suggest that similar principles could be applied to monitoring routines, particularly". It would be beneficial to include a few sentences about the state of monitoring routines in real-world settings, perhaps by citing a review involving ischemic heart disease and CHD as well as a novel TIA/stroke mood monitoring study (below). The range of remote monitoring digital tools should be discussed here.

#1 - Indraratna P, Tardo D, Yu J, Delbaere K, Brodie M, Lovell N, Ooi SY

Mobile Phone Technologies in the Management of Ischemic Heart Disease, Heart Failure, and Hypertension: Systematic Review and Meta-Analysis JMIR Mhealth Uhealth 2020;8(7):e16695 doi: 10.2196/16695

#2 - Zawada S, Acosta J, Collins C, Dumitrascu O, Harahsheh E, Hagen C, et al. Real-World Smartphone Data Predicts Mood After Ischemic Stroke and Transient Ischemic Attack Symptoms and May Constitute Digital Endpoints: A Proof-of-Concept Study. Mayo Clinic 598 Proceedings: Digital Health. 2025;3(3)

Response: We thank you for this thoughtful suggestion. However, our study specifically focused on physician-led, imaging-based monitoring in outpatient care. The integration of remote monitoring technologies lies outside the scope of our research. To maintain conceptual and methodological focus, we therefore decided not to expand the discussion in this direction.

Strengths and limitations - were all interviews conducted in patients' homes?

Response: We clarified the settings of the interviews in the strengths and limitations section.

Changes in manuscript: “Interviews were conducted either in participants’ homes, at one of the study centres, via online Zoom call, or via telephone, depending on participant preference and health status. This flexibility ensured accessibility and comfort for all participants.”
